# Supplementary material for: Joint control of meiotic crossover patterning by the synaptonemal complex and HEI10 dosage
Source: Nat Commun. 2022 Oct 12;13:5999. doi: 10.1038/s41467-022-33472-w (PMC9556546; doi:10.1038/s41467-022-33472-w)
Supplement: Supplementary file 1 — Supplementary Information [file 41467_2022_33472_MOESM1_ESM.pdf]

Supplementary Information

# Joint control of meiotic crossover patterning by the synaptonemal complex and HEI10 dosage

Stéphanie Durand[1*], Qichao Lian[1*], Juli Jing[1*], Marcel Ernst[2], Mathilde Grelon[3], David Zwicker[2] and Raphael Mercier[1#]

[*] Contributed equally to this work

[#] Corresponding author: Raphael Mercier (mercier@mpipz.mpg.de)

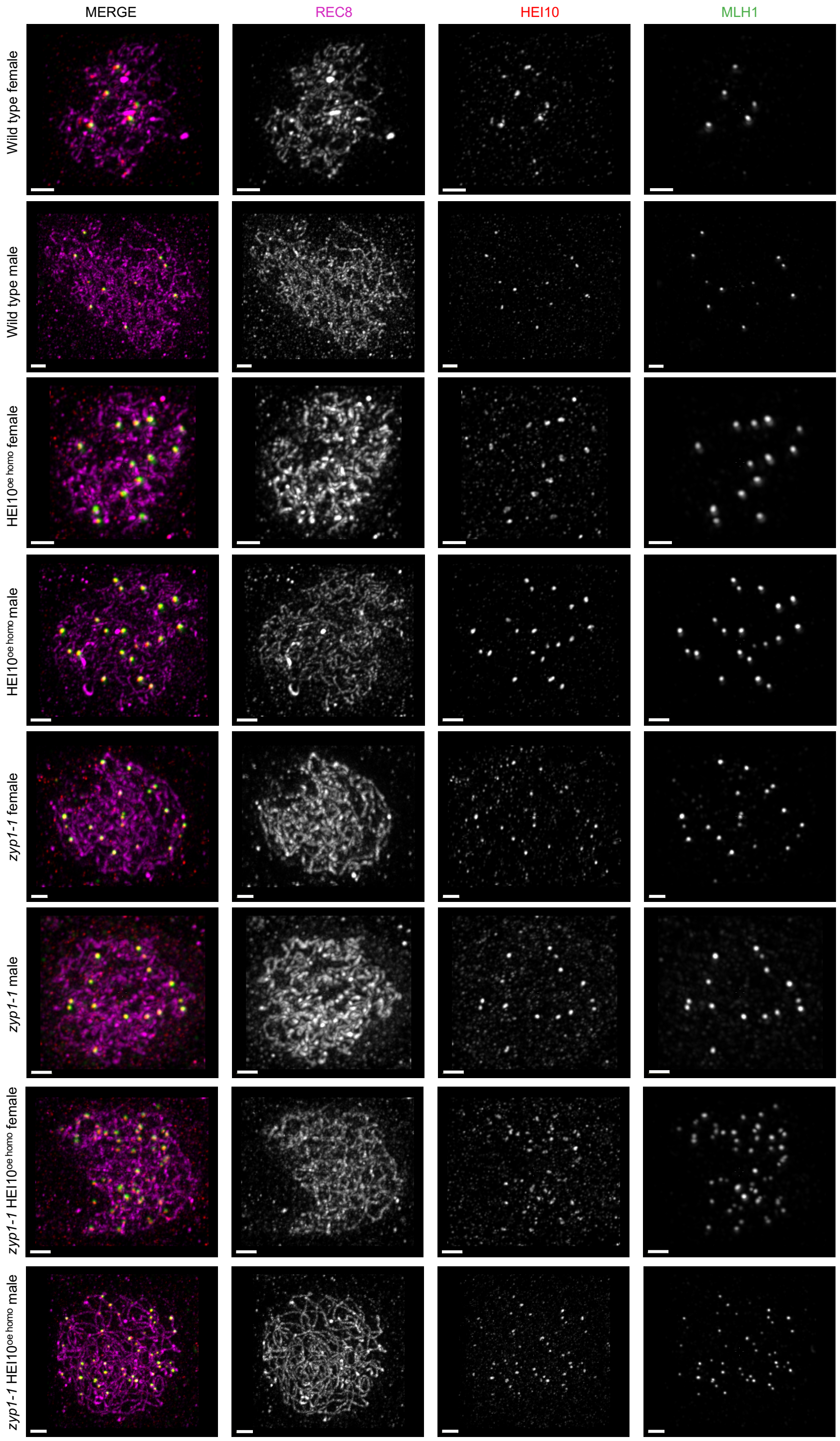

Supplementary Figure 1. Localization of REC8, MLH1 and HEI10 at diplotene.
Following immunolocalization, REC8 (Purple) and HEI10 (red) were imaged with STED while MLH1 (green) was imaged with confocal microscopy. The maximum intensity projection is shown. Scale bar=1μm

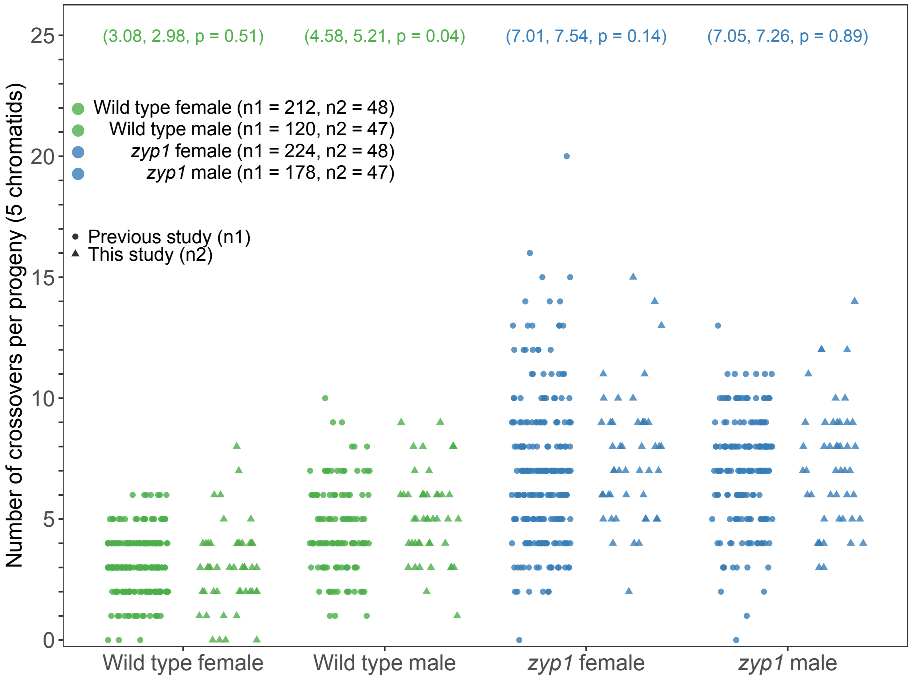

Supplementary Figure 2. Comparison of CO numbers between previous studies and this work.

The number of COs per transmitted chromatid in female and male of wild type and *zyp1,* from a previous study (Capilla et al, 2020 [8]), compared to this study. Different genotypes and studies are indicated through different colors and shapes, respectively. The mean CO number and *p*-value for differences (Two-sided Mann-Whitney test) are indicated at the top in parentheses, with the same color codes. Source data are provided as a Source Data file.

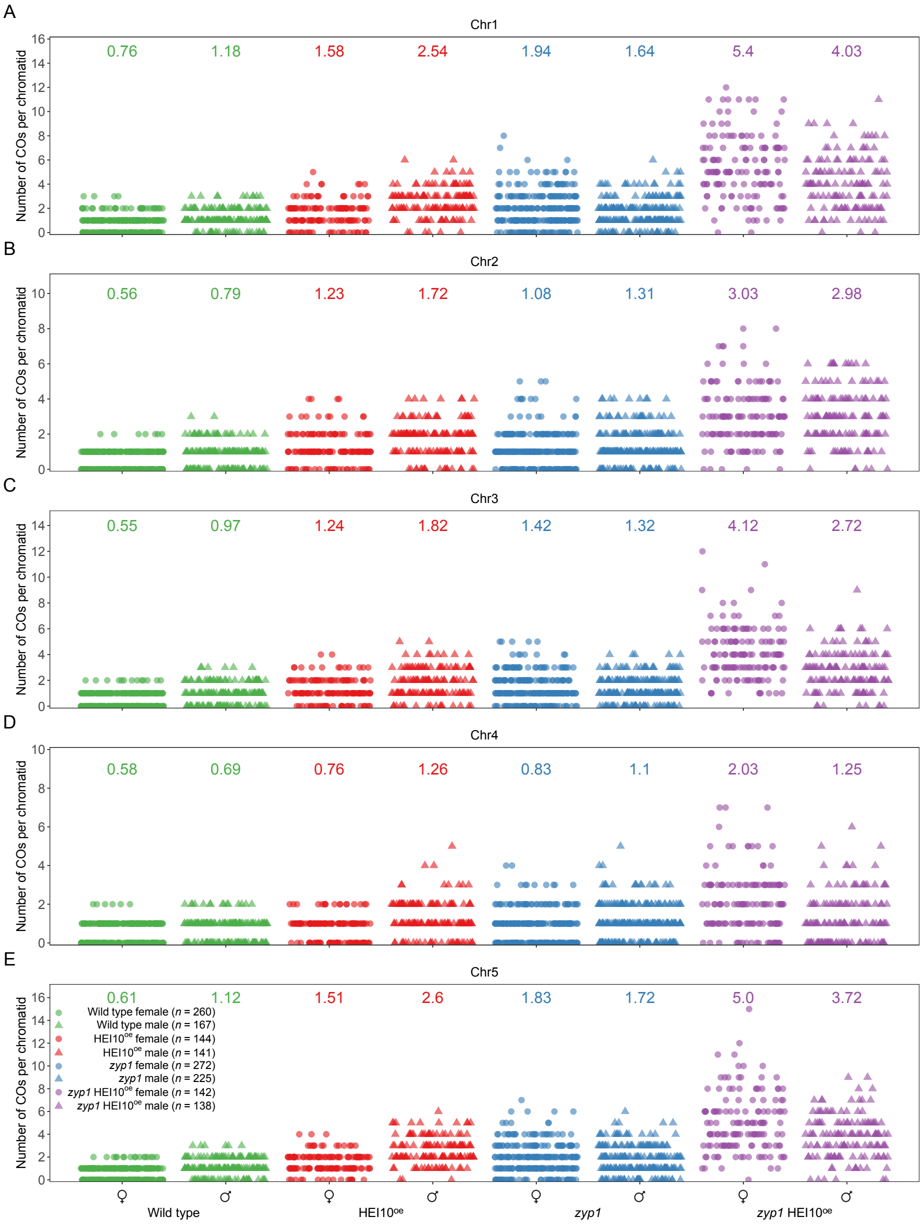

Supplementary Figure 3. CO numbers per chromosome.
The number of COs per transmitted chromatid in female and male populations of wild type (green), HEI10oe (red), *zyp1* (blue) and *zyp1* HEI10oe (purple), respectively. (A-E), chromosome one to five, respectively. The mean CO number of the population is indicated on the top, with the same color codes. Source data are provided as a Source Data file.

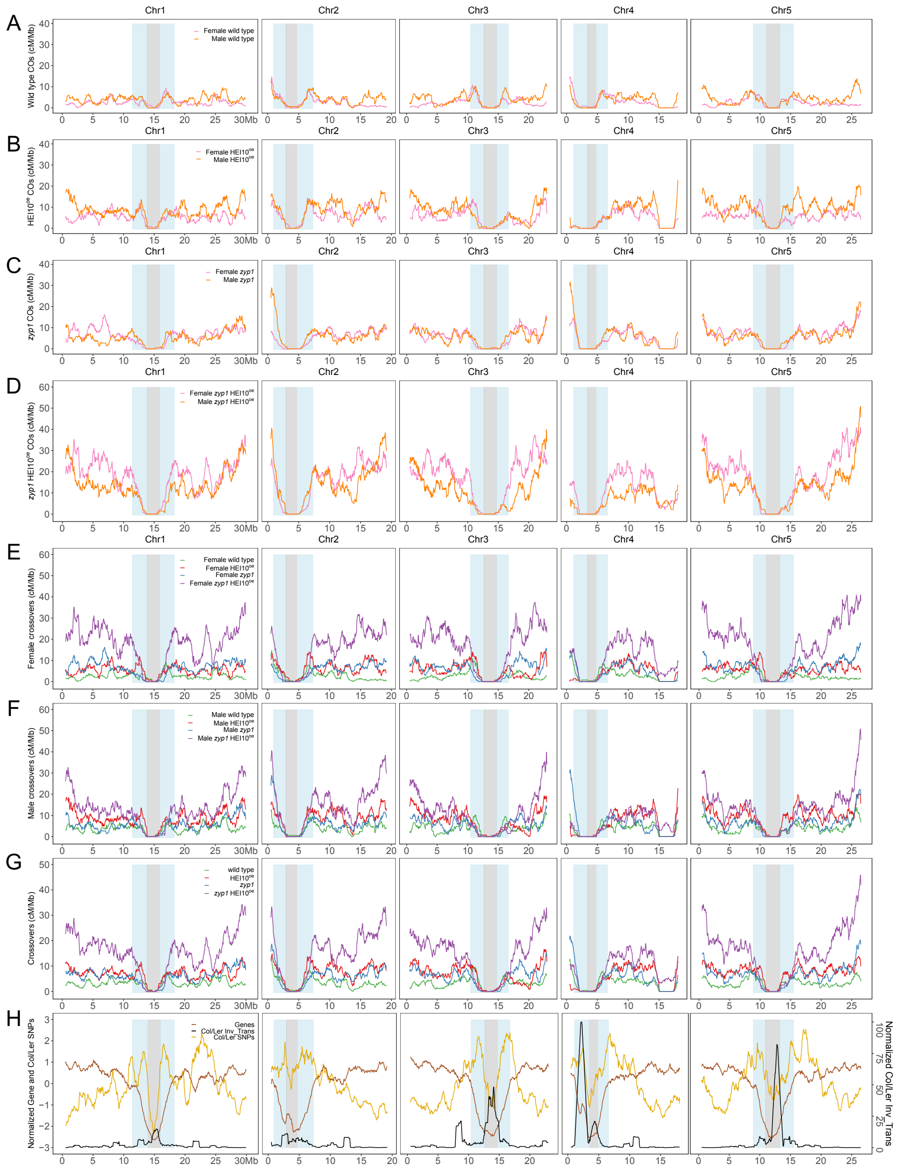

Supplementary Figure 4. The distribution of CO frequency along chromosomes.
Comparison of CO distributions (sliding window-based, with window size of 1 Mb and step size of 50 kb) between (A) female and male of wild type; (B) HEI10^{oe}; (C) *zyp1*; and (D) *zyp1* HEI10^{oe}. (E) Comparison of CO distributions in female. (F) Comparison of CO distributions in males (F). (G) Comparison of CO distribution (merged female and male) among populations. (H) Protein coding gene, SNP densities, and frequency of base pairs affected by inversions or translocations (>100bp) between Col and L*er* (See Methods), with 1 Mb window size and 50 kb step size. The pericentromeric and centromeric regions are indicated by grey and blue shading, respectively. Consistent with previous observations[8, 29], there is a ~2.2 Mb region on the long arm of chromosome 4 where recombination is suppressed, which suggests a structural arrangement between the Col and L*er* strains.

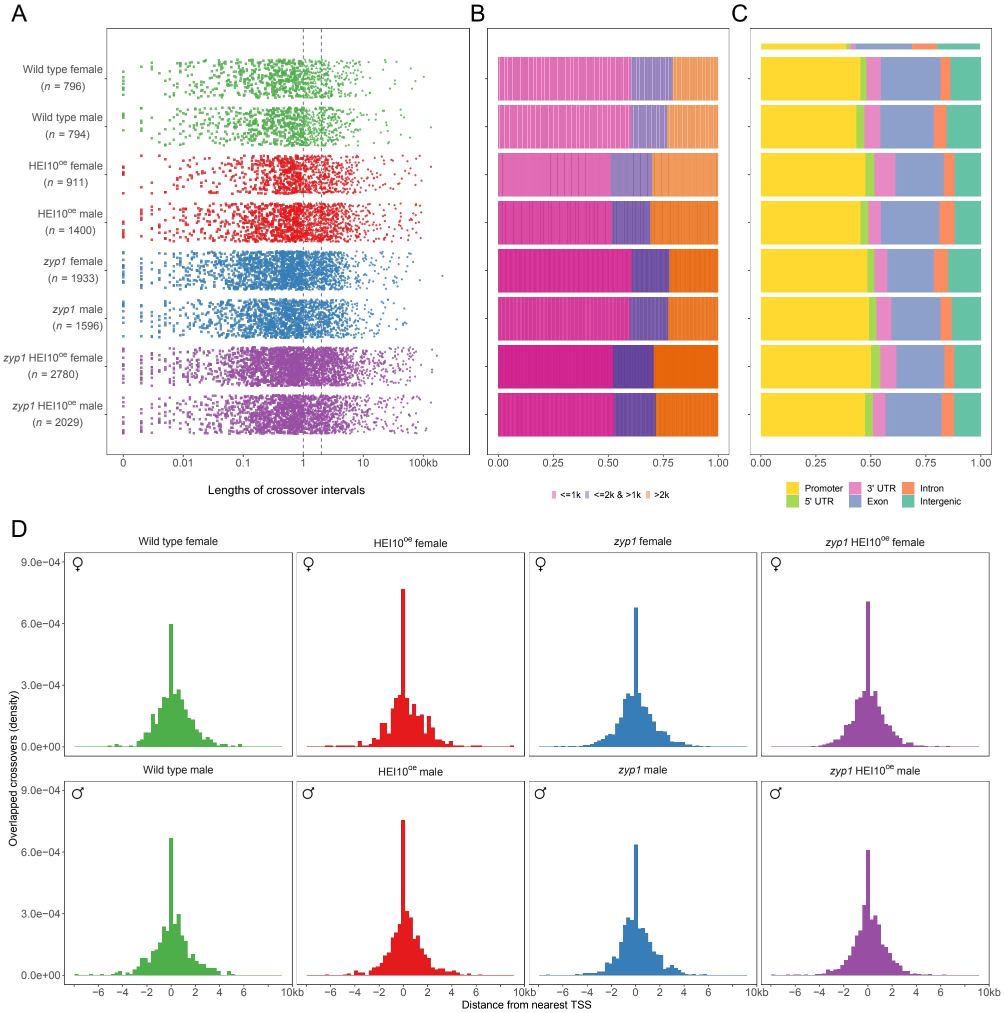

Supplementary Figure 5. The fine-scale distribution of COs.

(A) The distribution of the length of CO intervals across populations. The length of 1 kb and 2 kb are indicated by grey dashed lines. The number of analyzed COs is shown in parentheses. The median of CO intervals is 819 bp (B) The distribution of proportion of COs with interval lengths less than 1 kb (high-resolution), more than 2 kb, and the rest separately. (C) The distribution of proportion of high-resolution COs overlapping with genomic features. The promoter region is defined as the 2 kb upstream of the transcription start site. The proportion of the different genomic features is shown as the bar on the top, which is defined by following the priority of promoter, 5' UTR, 3' UTR, exon, intron, and intergenic regions. (D) The distribution of distance of high-resolution COs from nearest TSS.

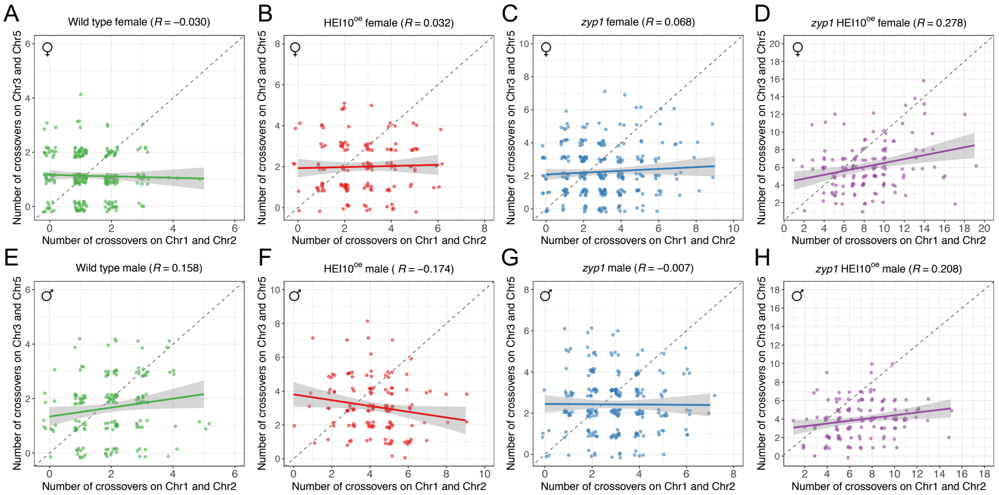

Supplementary Figure 6. Correlation analysis of CO numbers between chromosomes transmitted by the same gamete.

Pearson's correlation analysis of CO numbers between chromosomes in the same gamete was performed in female and male populations of wild type (A, E), HEI10^{oe} (B, F), *zyp1* (C, G), and *zyp1* HEI10^{oe} (D, H). The sum of COs detected on chromosomes 1 and 2 was plotted against the sum of COs on chromosomes 3 and 5 in the same gamete. A jitter function was applied to avoid overlapping points. The gray shading represents the 95% confidence interval. The correlation coefficients are shown in parentheses. The very low correlation contrasts observations in several other species[27] and may be due to lower cell-to-cell variation in Arabidopsis.

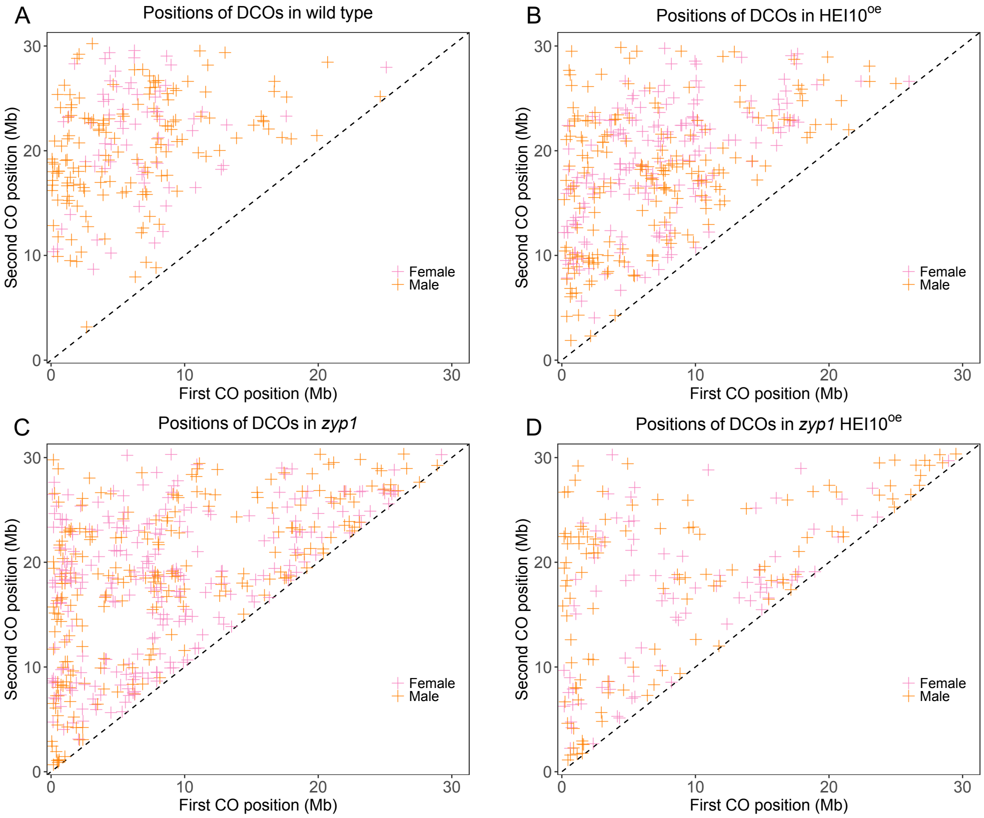

Supplementary Figure 7. The distribution of positions of double-COs.
The relative positions of COs for chromosomes with exactly two COs (as in figure 2C-F). The position of the first and second CO of the pair, in female and male meiosis of wild type (A), HEI10^{oe} (B), *zyp1* (C), and *zyp1* HEI10^{oe} (D), respectively. Source data are provided as a Source Data file.

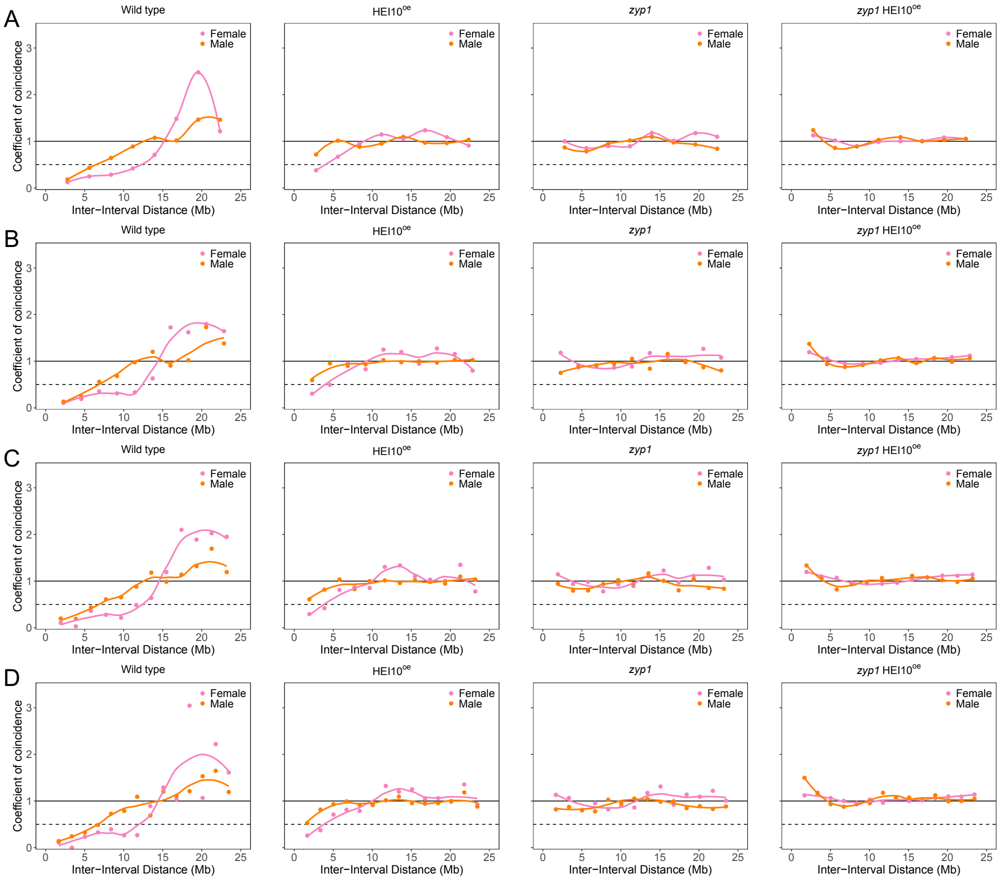

Supplementary Figure 8. The CoC analysis is robust to interval number variation

CoC curves in female and male meiosis of wild type, HEI10^{oe}, *zyp1*, and *zyp1* HEI10^{oe}, respectively. Chromosomes were divided into 9 (A), 11 (B), 13 (C) and 15 (D) intervals, for calculating the mean coefficient of coincidence of each pair of intervals.

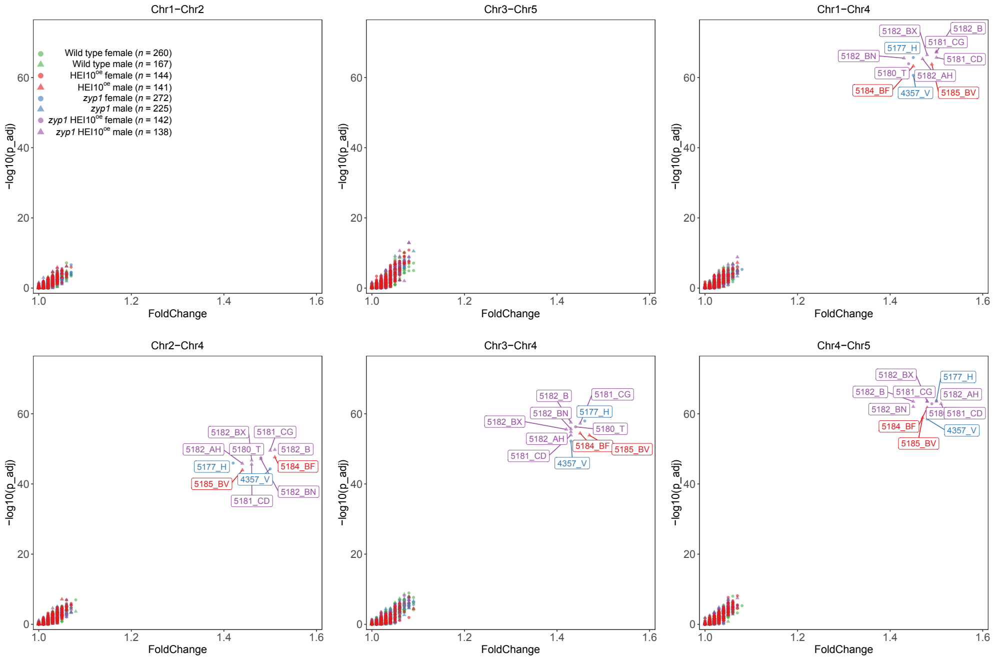

Supplementary Figure 9. The analysis of sequencing depths along chromosomes for screening aneuploidy.

The sequencing depth was calculated for each 100kb non-overlapped interval along chromosomes. The two-sided Mann-Whitney test was used for checking the difference between pair of chromosomes, the significant p value was then adjusted by fdr method. The name of the detected aneuploidy is shown with the same color-codes as for corresponding populations.

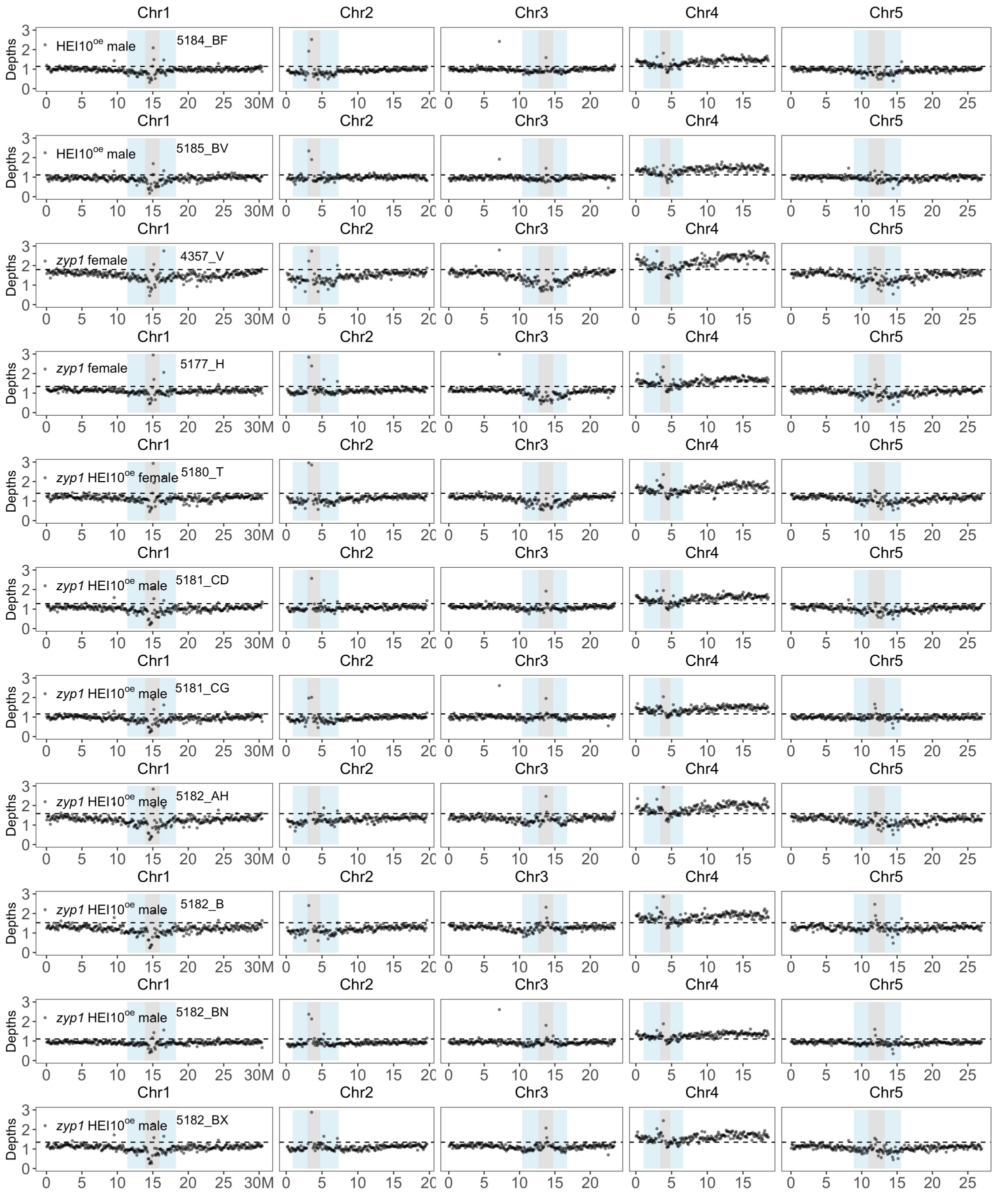

Supplementary Figure 10. Sequencing depth along aneuploid chromosomes.
The sequencing depth was calculated for each 100 kb non-overlapped interval along chromosomes. The pericentromeric and centromeric regions are indicated by grey and blue shading, respectively. The horizontal dashed line indicates the mean sequencing depth of the sample. Aneuploidy is visible by higher coverage of one chromosome compared to the others. The label of the detected aneuploidy and corresponding populations are presented individually.

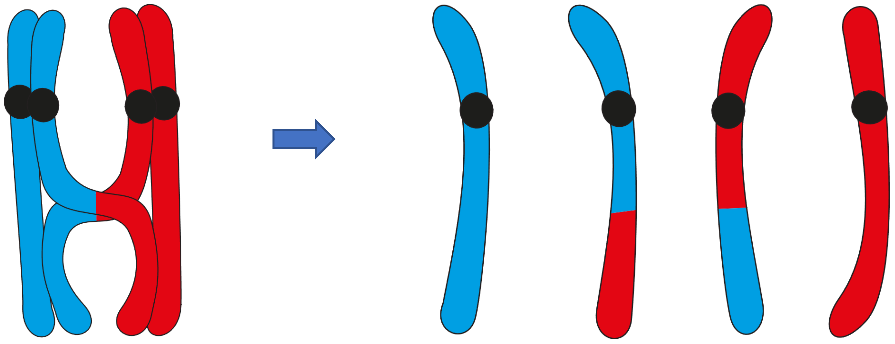

Supplementary Figure 11. Relationship between crossover per bivalent and crossover per transmitted chromatid

As a crossover affects two of the four chromatids of a bivalent (pair of homologous chromosomes), one crossover on a bivalent translates into half of the chromatid having one crossover, and half of the chromatid having zero crossovers
