# Supplementary material for: Joint control of meiotic crossover patterning by the synaptonemal complex and HEI10 dosage
Source: Nat Commun. 2022 Oct 12;13:5999. doi: 10.1038/s41467-022-33472-w (PMC9556546; doi:10.1038/s41467-022-33472-w)
Supplement: Supplementary file 3 — Description of Additional Supplementary Files [file 41467_2022_33472_MOESM3_ESM.pdf]

Description of Additional Supplementary Files

File name: Supplementary Data 1

Description: List of genetic crossovers and their positions, in female and male of wild type, zyp1, HEI10oe and zyp1 HEI10oe. Raw data source for Figure 1D-F, Figure 2, Figure 3N, Figure 3Q, Figure 4B, Supplementary Figure 2-8.

File name: Supplementary Data 2

Description: Genotyping primers used in this study.
