# Supplementary material for: Joint control of meiotic crossover patterning by the synaptonemal complex and HEI10 dosage
Source: Nat Commun. 2022 Oct 12;13:5999. doi: 10.1038/s41467-022-33472-w (PMC9556546; doi:10.1038/s41467-022-33472-w)
Supplement: Supplementary file 6 — Reporting Summary [file 41467_2022_33472_MOESM6_ESM.pdf]

nature portfolio

Corresponding author(s): Raphael Mercier

Last updated by author(s): 2022/09/12

Reporting Summary

Nature Portfolio wishes to improve the reproducibility of the work that we publish. This form provides structure for consistency and transparency in reporting. For further information on Nature Portfolio policies, see our Editorial Policies and the Editorial Policy Checklist.

Statistics

For all statistical analyses, confirm that the following items are present in the figure legend, table legend, main text, or Methods section.

n/a	Confirmed	
☐	☒	The exact sample size (*n*) for each experimental group/condition, given as a discrete number and unit of measurement
☐	☒	A statement on whether measurements were taken from distinct samples or whether the same sample was measured repeatedly
☐	☒	The statistical test(s) used AND whether they are one- or two-sided<br>*Only common tests should be described solely by name; describe more complex techniques in the Methods section.*
☐	☒	A description of all covariates tested
☐	☒	A description of any assumptions or corrections, such as tests of normality and adjustment for multiple comparisons
☐	☒	A full description of the statistical parameters including central tendency (e.g. means) or other basic estimates (e.g. regression coefficient) AND variation (e.g. standard deviation) or associated estimates of uncertainty (e.g. confidence intervals)
☐	☒	For null hypothesis testing, the test statistic (e.g. *F*, *t*, *r*) with confidence intervals, effect sizes, degrees of freedom and *P* value noted<br>*Give P values as exact values whenever suitable.*
☒	☐	For Bayesian analysis, information on the choice of priors and Markov chain Monte Carlo settings
☒	☐	For hierarchical and complex designs, identification of the appropriate level for tests and full reporting of outcomes
☐	☒	Estimates of effect sizes (e.g. Cohen's *d*, Pearson's *r*), indicating how they were calculated

Our web collection on statistics for biologists contains articles on many of the points above.

Software and code

Policy information about availability of computer code

Data collection	No software was used to collect data
Data analysis	Foci counts and SC length measurements were performed using Huygens essential 21.10.1p1 64b and Imaris 9.6.0 as described in the methods. Statistical tests were performed in Prism 9.4.1<br>Crossover analysis: All softwares and statistic tests used (described in the Methods) including: FastQC (v0.11.9), BWA (v0.7.15-r1140), Sambamba (v0.6.8), inGAP-family (v1.0.0), MADpattern, ChIPseeker (v1.22.1) and Mosdepth (v0.2.7), and R packages ggplot2 (v3.3.5) for the most of the visualization.

For manuscripts utilizing custom algorithms or software that are central to the research but not yet described in published literature, software must be made available to editors and reviewers. We strongly encourage code deposition in a community repository (e.g. GitHub). See the Nature Portfolio guidelines for submitting code & software for further information.

Data

Policy information about availability of data

All manuscripts must include a data availability statement. This statement should provide the following information, where applicable:

- Accession codes, unique identifiers, or web links for publicly available datasets
- A description of any restrictions on data availability
- For clinical datasets or third party data, please ensure that the statement adheres to our policy

Source data are provided with this paper. Raw MLH1 counts and SC length measurements are shown in source data file Figure 1B, and source data file Figure 4M, respectively. The list of identified COs in the female and male populations of wild type, HEI10oe, zyp1, and zyp1 HEI10oe can be accessed in supplementary dataset S1. The raw sequencing data generated in this study have been deposited in the ArrayExpress EMBL-EBI database under accession code E-MTAB-11696 (https://

www.ebi.ac.uk/arrayexpress/experiments/E-MTAB-11696). Reference Col (TAIR10) and Ler genomes used in this study can be found in https://www.arabidopsis.org/ and https://1001genomes.org/data/MPIPZ/MPIPZJiao2020/releases/current/strains/Ler/

Field-specific reporting

Please select the one below that is the best fit for your research. If you are not sure, read the appropriate sections before making your selection.

☒ Life sciences ☐ Behavioural & social sciences ☐ Ecological, evolutionary & environmental sciences

For a reference copy of the document with all sections, see nature.com/documents/nr-reporting-summary-flat.pdf

Life sciences study design

All studies must disclose on these points even when the disclosure is negative.

Sample size	No sample size calculation were performed as part of this study. Sample size for sequencing was chosen taking into account sequencing costs and aiming at ~200 per population, which allows meaningful analysis of crossover distribution along chromosomes. For cytology we aimed for 20 high quality cells per condition. The exact number may vary depending on the difficulty of the preparation (e.g in female)
Data exclusions	No data was excluded
Replication	At least three independent plants were used for each conditions.
Randomization	Randomization was not relevant to this study. Plants were assigned to different groups based on their genotype. As the different genotypes were identified in segregating populations, randomization is generated by the segregation of alleles in the population.
Blinding	The investigators were not blinded to group allocation. Blinding was not relevant to this study as the same computational analysis pipelines were used for wild type and mutants.

Reporting for specific materials, systems and methods

We require information from authors about some types of materials, experimental systems and methods used in many studies. Here, indicate whether each material, system or method listed is relevant to your study. If you are not sure if a list item applies to your research, read the appropriate section before selecting a response.

Materials & experimental systems

n/a	Involved in the study	
☐	☒	Antibodies
☒	☐	Eukaryotic cell lines
☒	☐	Palaeontology and archaeology
☐	☒	Animals and other organisms
☒	☐	Human research participants
☒	☐	Clinical data
☒	☐	Dual use research of concern

Methods

n/a	Involved in the study	
☒	☐	ChIP-seq
☒	☐	Flow cytometry
☒	☐	MRI-based neuroimaging

Antibodies

Antibodies used	Four primary antibodies were used: anti-REC8 raised in rat (ref 40) (laboratory code PAK036, dilution 1:250), anti-MLH1 in rabbit (ref41) (PAK017, 1:200), and anti-HEI10 in chicken (ref19) (PAK046, 1:5,000), Secondary antibodies (dilution 1:250) were Abberior STAR ORANGE Goat anti-rat IgG (catalog # STORANGE-1007), STAR RED Goat anti-chicken IgY (catalog # STRED-1005), STAR GREEN Goat anti-rabbit IgG (catalog # STGREEN-1002)
Validation	The primary antibodies used in this study were widely used and validated as part of previous publications:<br>anti-REC8: Cromer L, et al. . Current Biology 23, 2090-2099 (2013). Duroc et al, Plos Genetics e1004674 (2014), Jahns et al, Plos Biology e1001930 (2014), Cromer et al, PNAS116, 16018-16027 (2019); Capilla et al, PNAS e2023613118 (2021)<br>anti-MLH1: Chelysheva et al, Cytogenetic and genome research 129, 143-153 (2010). Chelysheva et al Plos Genet e1002799 (2012). Hurel et al, The Plant Journal 95(2) 385-396 (2018). Jahns et al, Plos Biology e1001930 (2014). Capilla et al, PNAS e2023613118 (2021)<br>Anti-HEI10: Chelysheva L, et al. PLoS Genet 8, e1002799 (2012). Capilla et al, PNAS e2023613118 (2021)

Animals and other organisms

Policy information about studies involving animals; ARRIVE guidelines recommended for reporting animal research

Laboratory animals	The study did no involved animals but laboratory plants.
Wild animals	The study did no involved animals.
Field-collected samples	*For laboratory work with field-collected samples, describe all relevant parameters such as housing, maintenance, temperature, photoperiod and end-of-experiment protocol OR state that the study did not involve samples collected from the field.*
Ethics oversight	*Identify the organization(s) that approved or provided guidance on the study protocol, OR state that no ethical approval or guidance was required and explain why not.*

Note that full information on the approval of the study protocol must also be provided in the manuscript.
